# *NRG1*, *PIP4K2A*, and *HTR2C* as Potential Candidate Biomarker Genes for Several Clinical Subphenotypes of Depression and Bipolar Disorder

**DOI:** 10.3389/fgene.2020.00936

**Published:** 2020-08-25

**Authors:** Anastasia Levchenko, Natalia M. Vyalova, Timur Nurgaliev, Ivan V. Pozhidaev, German G. Simutkin, Nikolay A. Bokhan, Svetlana A. Ivanova

**Affiliations:** ^1^Theodosius Dobzhansky Center for Genome Bioinformatics, Saint Petersburg State University, Saint Petersburg, Russia; ^2^Tomsk National Research Medical Center, Mental Health Research Institute, Russian Academy of Sciences, Tomsk, Russia; ^3^Institute of Translational Biomedicine, Saint Petersburg State University, Saint Petersburg, Russia; ^4^National Research Tomsk State University, Tomsk, Russia; ^5^Siberian State Medical University, Tomsk, Russia; ^6^National Research Tomsk Polytechnic University, Tomsk, Russia

**Keywords:** neuregulin 1, serotonin 2C receptor, phosphatidylinositol-5-phosphate 4-kinase type 2 alpha, depressive episode, bipolar affective disorder, treatment response, severity, time to recurrence

## Abstract

GSK3B, BDNF, NGF, NRG1, HTR2C, and PIP4K2A play important roles in molecular mechanisms of psychiatric disorders. GSK3B occupies a central position in these molecular mechanisms and is also modulated by psychotropic drugs. BDNF regulates a number of key aspects in neurodevelopment and synaptic plasticity. NGF exerts a trophic action and is implicated in cerebral alterations associated with psychiatric disorders. NRG1 is active in neural development, synaptic plasticity, and neurotransmission. HTR2C is another important psychopharmacological target. PIP4K2A catalyzes the phosphorylation of PI5P to form PIP2, the latter being implicated in various aspects of neuronal signal transduction. In the present study, the six genes were sequenced in a cohort of 19 patients with bipolar affective disorder, 41 patients with recurrent depressive disorder, and 55 patients with depressive episode. The study revealed a number of genetic variants associated with antidepressant treatment response, time to recurrence of episodes, and depression severity. Namely, alleles of rs35641374 and rs10508649 (*NRG1* and *PIP4K2A*) may be prognostic biomarkers of time to recurrence of depressive and manic/mixed episodes among patients with bipolar affective disorder. Alleles of NC_000008.11:g.32614509_32614510del, rs61731109, and rs10508649 (also *NRG1* and *PIP4K2A*) seem to be predictive biomarkers of response to pharmacological antidepressant treatment on the 28th day assessed by the HDRS-17 or CGI-I scale. In particular, the allele G of rs10508649 (*PIP4K2A*) may increase resistance to antidepressant treatment and be at the same time protective against recurrent manic/mixed episodes. These results support previous data indicating a biological link between resistance to antidepressant treatment and mania. Bioinformatic functional annotation of associated variants revealed possible impact for transcriptional regulation of *PIP4K2A*. In addition, the allele A of rs2248440 (*HTR2C*) may be a prognostic biomarker of depression severity. This allele decreases expression of the neighboring immune system gene *IL13RA2* in the putamen according to the GTEx portal. The variant rs2248440 is near rs6318 (previously associated with depression and effects of psychotropic drugs) that is an eQTL for the same gene and tissue. Finally, the study points to several protein interactions relevant in the pathogenesis of mood disorders. Functional studies using cellular or animal models are warranted to support these results.

## Introduction

*GSK3B* (Beaulieu, 2012), *BDNF* (Lu et al., 2014), *NGF* (Cirulli and Alleva, 2009), *NRG1* (Mei and Nave, 2014), *HTR2C* (Chagraoui et al., 2016; Palacios et al., 2017), and *PIP4K2A* (Wilcox and Hinchliffe, 2008) are genes that showed some importance in the study of the molecular etiology of psychiatric disorders (Table 1). Specifically, *GSK3B* that codes for glycogen synthase kinase 3β plays a central role in the pathogenesis of psychiatric disorders (Beaulieu, 2012; Emamian, 2012). This gene is directly or indirectly inhibited by antidepressants, lithium, and antipsychotics (Beaulieu et al., 2009; Freyberg et al., 2010). It is furthermore associated with response to antidepressant medication in patients with depressive disorders (Tsai et al., 2008; Levchenko et al., 2018) and lithium treatment in patients with bipolar disorder (Benedetti et al., 2005; Lin et al., 2013; Iwahashi et al., 2014; Benedetti et al., 2015).

**TABLE 1:** Previous reports of association between the six sequenced genes and psychiatric phenotypes, including response to mediation.

Genes	Variants	Associated phenotypes	References
*GSK3B*	rs6438552	Brain structural changes in major depressive disorder (MDD)	Inkster et al., 2009
	rs6438552 rs334558	Age of onset of bipolar disorder (BD) in female patients Response to lithium treatment	Lin et al., 2013
	Haplotype containing rs334558 and rs3755557	Response to lithium treatment	Iwahashi et al., 2014
	rs334558	Response to lithium augmentation	Adli et al., 2007
	rs334558	Gray matter volumes in the right frontal lobe of patients with BD	Benedetti et al., 2015
	rs334558	Remission in patients with depressive disorders	Levchenko et al., 2018
	rs334558, rs13321783, rs2319398	Response to antidepressant therapy	Tsai et al., 2008
	rs12630592	Severity of mania (in both acute and stabilized periods) and depression in stabilized periods	Bureau et al., 2017
	Haplotype containing rs334555, rs119258668, and rs11927974	Age of onset of MDD	Saus et al., 2010
	Interaction of rs6782799, rs6265 (*BDNF*), and negative life events	MDD	Yang et al., 2010
*BDNF*	rs6265	MDD	Tsai et al., 2003; Schroter et al., 2020
	Interaction of rs6265 and stressful life events	MDD	Hosang et al., 2014
	rs6265	Recurrent MDD	Xiao et al., 2011; Lee Y. et al., 2013
	rs6265	Severity of depression	Losenkov et al., 2020
	rs6265	Response to antidepressant therapy and remission in MDD	Choi et al., 2006; Alexopoulos et al., 2010; Chi et al., 2010; Zou et al., 2010; Taylor et al., 2011; Murphy et al., 2013; Wang et al., 2014; Colle et al., 2015; Xin et al., 2020
	rs712442	Response to antidepressant therapy	Ochi et al., 2019
*NGF*	Haplotype containing rs2254527, rs6678788, and rs12760036	Remission rate in MDD	Yeh et al., 2015
	Interaction with *BDNF*, among 590 other polygenes	Suicide attempt	Sokolowski et al., 2016
*NRG1*	rs4733272 (together with other chromosome 8-associated SNPs)	Schizophrenia, BD and MDD	Chen et al., 2017
	rs4236710 and rs4512342; haplotype containing rs4512342 and rs6982890 rs2919375 Haplotype containing rs4531002 and rs11989919	Schizophrenia MDD MDD and BD	Wen et al., 2016
	rs35753505 and rs7014762	BD	Mei and Nave, 2014
	rs6994992, rs2439272, rs62510682, rs10503929, and rs3924999	Prepulse inhibition (PPI, a measure of inhibitory sensorimotor gating)	Hong et al., 2008; Roussos et al., 2011
	rs6994992	Activity of frontal and temporal lobes, premorbid IQ, and positive symptoms in schizophrenia	Hall et al., 2006; Papiol et al., 2011
	rs3924999	Perceptual aberrations in schizotypal personality disorder	Lin et al., 2005
*HTR2C*	rs6318	Severity of depression	Brummett et al., 2014
	rs6318	Response to antidepressant therapy	Vyalova et al., 2017
	rs6318	Suicide attempt	Karanovic et al., 2015
	rs6318	Dysregulated stress responding and risk for depression	Avery and Vrshek-Schallhorn, 2016
	rs6318	Stress-induced mesoaccumbal dopamine release	Mickey et al., 2012
	rs6318 and rs3813929	Feeding behavior and antipsychotics-induced weight gain and movement disorders	Drago and Serretti, 2009
	rs1414334	Metabolic syndrome in patients using antipsychotics	Mulder et al., 2009; Risselada et al., 2012
*PIP4K2A*	rs10828317	Tardive dyskinesia in schizophrenia patients	Fedorenko et al., 2014
	rs10828317	Schizophrenia	Schwab et al., 2006; Bakker et al., 2007; Fedorenko et al., 2013
	rs10828317 and rs10430590	CGI-S total score at day 28 of antidepressant therapy	Vyalova et al., 2017
	rs11013052	Schizophrenia	Saggers-Gray et al., 2008
	rs8341	Schizophrenia	He et al., 2007
	Various intronic deletions and insertions 29 bp from the exon 9–intron 9 junction	BD	Stopkova et al., 2003

*BDNF* codes for Brain-Derived Neurotrophic Factor, active in neurodevelopment and synaptic plasticity (Begni et al., 2017; Numakawa et al., 2018); the gene has been extensively studied in the context of psychiatric disorders (Numakawa et al., 2018). In patients with depression, BDNF levels are decreased and methylation levels in the gene’s promoter are increased, indicating attenuated gene expression (Kishi et al., 2017; Schroter et al., 2019). Variants in this gene are associated with severity of depression in drug-naive depressed patients (Losenkov et al., 2020) and with drug response in patients who were taking antidepressant medication for the first time (Ochi et al., 2019).

*NGF* codes for nerve growth factor that exerts a trophic action on the cholinergic neurons of the basal forebrain nuclei and is implicated in cerebral alterations associated with psychiatric disorders (Bersani et al., 2000). Levels of this neurotrophin are decreased in bipolar disorder (Barbosa et al., 2011) and schizophrenia (Qin et al., 2017).

*NRG1*, coding for neuregulin, is implicated in neural development, including myelination, synaptic plasticity, and neurotransmission (Mei and Nave, 2014). It is associated with schizophrenia, bipolar disorder, and depression (Mei and Nave, 2014; Dang et al., 2016; Wen et al., 2016; Chen et al., 2017).

*HTR2C* codes for the serotonin 2C receptor that is an extremely important target of drugs used to treat a number of psychiatric disorders (Di Giovanni and De Deurwaerdere, 2016;

Palacios et al., 2017), including depression (Chagraoui et al., 2016). In addition, this gene is associated with severity of depression and response to pharmacological antidepressant treatment (Brummett et al., 2014; Vyalova et al., 2017).

*PIP4K2A* codes for phosphatidylinositol-5-phosphate 4-kinase type 2α, a kinase that catalyzes the phosphorylation of phosphatidylinositol-5-phosphate (PI5P) to form phosphatidylinositol-5,4-bisphosphate (PIP2) (Wilcox and Hinchliffe, 2008). The lipid PIP2 is implicated in various aspects of neuronal signal transduction (Di Paolo et al., 2004; Fedorenko et al., 2008; Fedorenko et al., 2009; Seebohm et al., 2014). Genetic studies of *PIP4K2A* showed an association with bipolar disorder and schizophrenia (Stopkova et al., 2003; Schwab et al., 2006), as well as with response to pharmacological antidepressant treatment (Vyalova et al., 2017). In addition, the mechanisms underlying lithium’s therapeutic efficacy in the chronic treatment of bipolar disorder include differential expression of *PIP4K2A* (Seelan et al., 2008).

**FIGURE 1:**
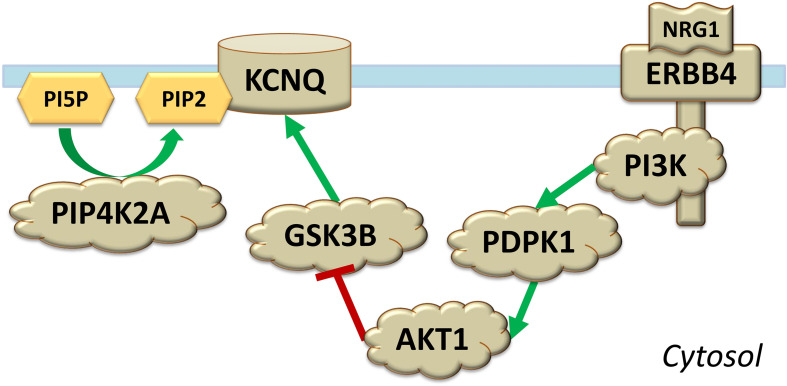
Shown are molecular interactions involving protein products of *PIP4K2A* and *NRG1* associated with time to recurrence of an episode in bipolar disorder and antidepressant treatment response. Phospholipids phosphatidylinositol-5-phosphate (PI5P) and phosphatidylinositol-5,4-bisphosphate (PIP2) are located on the intracellular leaflet of the plasma membrane, while the channel KCNQ and the receptor ERBB4 are transmembrane. The binding of NRG1 to the receptor tyrosine kinase ERBB4 results in subsequent activation of phosphoinositide 3-kinase (PI3K), 3-phosphoinositide-dependent protein kinase-1 (PDPK1), and AKT1; the latter kinase inhibits GSK3B.

*GSK3B*, *BDNF*, *NGF*, *NRG1*, *HTR2C*, and *PIP4K2A* are functionally connected. GSK3B plays a central role in the AKT/GSK3 molecular pathway (Beaulieu, 2012). The binding of either BDNF, NGF, or NRG1 to their respective receptor tyrosine kinases (TrkB, encoded by *NTRK2*, TrkA, encoded by *NTRK1*, and ERBB4, encoded by the gene with the same name) leads to inhibition of GSK3B via activation of phosphoinositide 3-kinase (PI3K), 3-phosphoinositide-dependent protein kinase-1 (PDPK1), and AKT1 (Beaulieu, 2012; Kim et al., 2014; Mei and Nave, 2014; Numakawa et al., 2018). PI3K is a heterodimer made of a catalytic p110 subunit and an adapter regulatory p85 subunit; in the brain, the former is encoded by *PIK3CA* and *PIK3CB*, while the latter is encoded by *PIK3R1* and *PIK3R2* (Dwivedi et al., 2008). Binding of serotonin to HTR2C leads to the opposite effect: GSK3B activation (Beaulieu, 2012). A link between PIP4K2A and GSK3B involves activation of M-channels by PIP2 and GSK3B (Figure 1; Delmas and Brown, 2005; Carter, 2007; Fedorenko et al., 2008; Wildburger and Laezza, 2012; Jiang et al., 2015). These channels, formed in the brain by tetramers of subunits potassium voltage-gated channel subfamily Q members 2, 3, and 5 (KCNQ2, KCNQ3, and KCNQ5), play a critical role in the regulation of neuronal excitability (Greene and Hoshi, 2017) and are potential treatment targets for manic symptoms (Grunnet et al., 2014).

In the present study, we sequenced exons and flanking intronic regions of *GSK3B, BDNF*, *NGF*, *NRG1*, *HTR2C*, and *PIP4K2A*, using the ion semiconductor next-generation sequencing technology. A statistical evaluation revealed that several alleles of *NRG1*, *HTR2C*, and *PIP4K2A* are associated with a number of clinical subphenotypes of depression and bipolar disorder, namely, time to recurrence of a depressive episode and of manic or mixed episode, response to pharmacological antidepressant treatment on the 28th day assessed by the Hamilton Depression Rating Scale—17 items (HDRS-17) or the Clinical Global Impression – Improvement (CGI-I) scale, and severity of depression. A bioinformatic assessment of the functional impact of these alleles suggests possible molecular mechanisms in the etiology of these clinical subphenotypes.

## Materials and Methods

### Study Subjects

The genetic study was carried out in accordance with The Code of Ethics of the World Medical Association (Declaration of Helsinki 1975, revised in Fortaleza, Brazil, 2013) for experiments involving humans. After approval of the study protocol by the Local Bioethics Committee of the Mental Health Research Institute in Tomsk, Russia, 115 patients were recruited from an in-patient facility of the same institute. As a negative control, 34 individuals without psychiatric disorders were also recruited into the study. Only subjects with European ancestry were considered. All participants gave written informed consent after a proper explanation of the prospective study.

In particular, we included the following numbers of patients with mood disorders, diagnosed using the criteria of the International Statistical Classification of Diseases and Related Health Problems 10th Revision (ICD-10): 19 patients with bipolar affective disorder (ICD-10: F31), 55 patients with depressive episode (ICD-10: F32), and 41 patients with recurrent depressive disorder (ICD-10: F33). A summary of clinical features for this cohort is shown in Table 2. Bipolar I and II disorders among patients with bipolar affective disorder were established using the Diagnostic and Statistical Manual of Mental Disorders, 4th edition, criteria. Severity of illness was assessed using the Clinical Global Impression—Severity scale (CGI-S) (Guy, 1976). Manic and mixed episodes were considered in the association study under a single category, because some patients only had depressive and mixed episodes. The 115 patients taken together constituted the cross-disorder group.

**TABLE 2:** Summary of clinical features of the cohort.

Bipolar affective disorder		nb of individuals/mean and standard deviation
Type	Bipolar I disorder	9
	Bipolar II disorder	10
Disease severity	Mild	7
	Moderate	7
	Severe	5
	Age of onset	30.42 ± 12.46
	Disease duration	10.32 ± 7.23
Total number of episodes	Depressive episodes	4.26 ± 3.33
	Manic and mixed episodes	7.42 ± 13.22
Gender	Male	8
	Female	11
**Depression**		**nb of individuals/mean and standard deviation**
Diagnosis	Recurrent depressive disorder	41
	Depressive episode	55
Syndrome	Depressive	45
	Anxious-depressive	42
	Asteno-depressive	9
Disease severity	Mild	0
	Moderate	86
	Severe	10
	Age of onset	45.84 ± 11.37
	Disease duration	4.59 ± 7.19
	Total number of episodes	2.29 ± 2.09
Gender	Male	16
	Female	80
HDRS-17	Response*	93
	No response	3
	Remission*	64
	No remission	32
CGI-I	Response*	91
	No response	5
CGI-S	Remission*	67
	No remission	29
	**_**	**nb of individuals/mean and standard deviation**
	Controls	34
	Age	29.44 ± 8.14
Gender	Male	11
	Female	23

* Response to/remission following pharmacological antidepressant treatment.

The duration of antidepressant treatment was not less than 28 days. During their follow-up in the clinic, patients were given several different groups of antidepressants, including tricyclic antidepressants, selective serotonin reuptake inhibitors, serotonin–norepinephrine reuptake inhibitors, noradrenergic and specific serotonergic antidepressants, and agomelatine (an agonist at melatonin receptors and an antagonist at serotonin 2C receptors). All antidepressants were used in recommended average therapeutic doses. For definition of response and remission, the HDRS-17 (Hamilton, 1960) and CGI scale (Guy, 1976) was used. Evaluation was made on the 28th day of treatment. Responders were identified if the HDRS-17 scores were reduced by 50% or if the CGI-I scores were ≤ 2. Remitters were identified if the HDRS-17 scores ≤ 7 or if the CGI-S scores were ≤ 2.

### Targeted DNA Sequencing

Evacuated blood collection tubes “Vacutainer” (Becton Dickinson, Franklin Lakes, NJ, United States) with EDTA as the anticoagulant were used. Extraction of DNA from whole venous blood was performed using the phenol–chloroform method. Concentration and purity of DNA were measured using the NanoDrop 8000 UV-Vis spectrophotometer (Thermo Fisher Scientific, Waltham, MA, United States).

Targeted next-generation sequencing was performed using the Ion Torrent semiconductor technology (Thermo Fisher Scientific, Waltham, MA, United States) at the Center “Medical Genomics” of the Tomsk National Research Medical Center. A custom DNA panel of 86 amplicons, covering 57 targets, was designed using the Ion AmpliSeq Designer^1^. The targets comprised exons and flanking intronic regions of at least 50 base pairs (bp) of *GSK3B*, *BDNF*, *NRG1*, *NGF*, *HTR2C*, and *PIP4K2A* in the human genome assembly GRCh37/hg19. The Ion AmpliSeq DNA Library kit 2.0 and the Ion Xpress Barcode Adapters 1–16 and 17–32 kits were used to prepare amplicon libraries based on the custom Ion AmpliSeq panels. Library normalization was performed using the Ion Library Equalizer kit. The Ion PGM Template OT2 200 Kit was used to prepare the template on the Ion OneTouch 2 System. Sequencing was done with the mean coverage of 473 × on the Ion Torrent Personal Genome Machine (PGM) System, using the Ion PGM Sequencing 200 kit v2 and Ion 316 Chips v2.

### Sequencing Analysis Workflow

The unmapped BAM files were converted to the FASTQ format using Picard tools^2^. Quality control (QC) of raw sequencing data and trimming of low-quality bases and adapters was done using FastQC^3^ and Trimmomatic (Bolger et al., 2014), respectively. The following Trimmomatic options were used: SLIDINGWINDOW: 4:20; LEADING: 15; TRAILING: 15; MINLEN: 36. QC of trimmed reads was also done using FastQC. Reads were aligned to the human genome assembly GRCh38.p12/hg38 using the default options of BWA-MEM^4^. SAMtools (Li et al., 2009) flagstat indicated that 99.84% of reads were aligned. SAMtools view with options -F 4 and –Sb, SAMtools sort, and SAMtools index were run to obtain indexed BAM files.

Discovery of single-nucleotide variants (SNV) and short indels was done using GATK (version 4.1.2.0) (McKenna et al., 2010)^5^. Genomic coordinates were lifted from the hg19 to hg38 assembly using the Lift Genome Annotations tool^6^. GATK HaplotypeCaller option stand-call-conf 20 was used. Variants were annotated with rsIDs listed in the Database of Single Nucleotide Polymorphisms build 153 (dbSNP)^7^, the Exome Aggregation Consortium (ExAC)^8^, and the Genome Aggregation Database (gnomAD)^9^.

In order to assure the extraction of reliable sequencing results, we used hard filtering parameters (DePristo et al., 2011; Van der Auwera et al., 2013)^10^. First, the following GATK VariantsFiltration cutoff was applied: QUAL < 100.0. Variants, which were mostly indels, called with this filter in ten or more samples, but not listed in the three databases, were considered ion semiconductor technology artifacts (Laehnemann et al., 2016) and removed. Next, the following GATK VariantsFiltration cutoffs were applied to the remaining variants: QD < 2.0, FS > 200.0, MQ < 40.0, MQRankSum < -12.5, ReadPosRankSum < -8. Males were considered as homozygotes for variants on chromosome X. PLINK 2.0^11^ was used to make the output files .bed, .bim, and .fam for the upcoming association study.

### Functional Annotation of Variants

Functional annotation of all discovered variants was done with ANNOVAR (Wang et al., 2010) that estimates the degree of deleteriousness of coding variants on protein function using 21 different prediction algorithms/conservation scores, SnpEff (Cingolani et al., 2012) (version 4.3) that was used to predict deleterious effects of both coding and non-coding variants (including effects of intronic variants on consensus sequences for splicing), HumanSplicingFinder (Desmet et al., 2009) that was used to predict impact of exonic variants on splicing, the GeneCards database (Stelzer et al., 2016) that lists GeneHancer (Fishilevich et al., 2017) regulatory elements for genes (for the purposes of this study, we considered only Elite GeneHancer elements), and the Genotype-Tissue Expression (GTEx) portal^12^ that lists expression quantitative trait loci (eQTLs) in various tissues. The GTEx Project was supported by the Common Fund of the Office of the Director of the National Institutes of Health, and by NCI, NHGRI, NHLBI, NIDA, NIMH, and NINDS. The data used for the analyses described in this manuscript were obtained from the GTEx Portal on 10/16/2019.

### Association Study

Tests under a number of statistical models were run to evaluate the association between SNVs or indels, discovered during sequencing, and clinical subphenotypes. For analysis of quantitative data (time to recurrence of an episode, age of onset) linear regression was used, whereas for binary data (response to treatment, remission following treatment) the statistical model was binomial logistic regression. These two types of tests were carried out using a series of applications that run in the R environment: snpStats (Sole et al., 2006), SNPRelate^13^, dplyr^14^, GenABEL^15^, ggplot2^16^, manhattanly^17^, GWASTools^18^, and GENESIS^19^. Multiple comparisons were dealt with using the Bonferroni correction and by controlling the false discovery rate (FDR). The type I error rate in this case was set to 5% (i.e., Bonferroni- and/or FDR-corrected *p*-values were considered significant when ≤ 0.05).

To evaluate the association between genetic variants and multinomial data (three disorders vs. controls, bipolar I and II disorders vs. controls, syndromes among patients with depression), multinomial logistic regression was deployed, while for ordinal data (severity of disease) the model was ordinal logistic regression. These last two types of statistical tests were carried out using Trinculo, a program run in the C++ environment that evaluates likelihood ratios within Bayesian and frequentist frameworks (Jostins and McVean, 2016). The type I error rate was set to 1% (i.e., likelihood ratio *p*-values were considered significant when ≤ 0.01).

## Results

### Sequencing Analysis

Sequencing analysis that included the indicated parameters of hard filtering resulted in 149 variant call format (VCF) files with 49 different variants, of which eight are novel, i.e., not listed in any of the three databases – dbSNP, ExAC, or gnomAD. Among the novel variants, four are 2–81 bp deletions, one is a 1 bp duplication, and three are 1 bp substitutions (Table 3). The novel variants (all in a heterozygous state, except for the 81 bp deletion present on the X chromosome in a male) were each present in one to three patients with depression, indicating minor allele frequency from 0.7 to 2% in the population under study.

**TABLE 3:** Novel variants discovered by sequencing.

Chr	Pos (hg38)	Ref	Alt	Variant	Gene	Strand	Possible function	nb of chr	Carriers
3	119947358	A	G	NC_000003.12:g. 119947358A > G	*GSK3B*	R	Intronic, no obvious function	3	d31, d138, d140
8	32614509-32614510	TT	del	NC_000008.11:g. 32614509_32614510del	*NRG1*	F		2	d95, d129
	32756363	A	dup	NC_000008.11:g. 32756363dup				1	d61
	32763214	T	G	NC_000008.11:g. 32763214T > G				2	d59, d128
10	22539905-22539911	GAGAGAG	del	NC_000010.11:g. 22539905_22539911del	*PIP4K2A*	R		1	d104
	22539924-22539937	AGAGAGAGGGAGAG	del	NC_000010.11:g. 22539924_22539937del				2	d18, d87
11	27658302	T	C	NC_000011.10:g. 27658302T > C	*BDNF*	R	Missense in all transcripts, Asp > Gly (acidic to neutral non-polar); deleterious/damaging (†ANNOVAR); allele G (reverse compliment) may affect splicing of the last intron of all *BDNF*’s transcripts, by erasing an exonic splicing enhancer motif (†HumanSplicingFinder)	1	d99
X	114906768-114906848	CAAGCTTTGATGTTACTGC ACGGCCACACCGAGG AACCGCCTGGACTAAGTCT GGATTTCCTGAA GTGCTGCAAGAGGAAT	del	NC_000023.11:g. 114906768_1149 06848del	*HTR2C*	F	Inframe deletion of 27 aa or frameshift deletion of 27 aa, resulting in 1 aa inserted; deleterious (†PROVEAN); may affect splicing of the last intron of all *HTR2C*’s transcripts, by erasing multiple exonic splicing enhancer motifs (†HumanSplicingFinder)	1	d128

*Chr, chromosome; pos, position in the human genome; ref, reference allele; alt, alternate allele; ^†^used algorithms.*

### Predicted Biological Impact of Discovered Variants

The results of functional annotation with SnpEff did not indicate high impact of either variant. Likewise, neither variant according to ANNOVAR is considered damaging/deleterious by all 21 prediction algorithms/conservation scores. HumanSplicingFinder indicated a number of new or disrupted exonic splicing enhancers (ESE) and exonic splicing silencers (ESS) (we show results only for novel and associated variants in section “Further Analysis of Biological Function of Associated and Novel Variants”). According to the list of Elite GeneHancer regulatory elements, both rs66866077 and rs79679324 (polymorphic in the cohort under study, but not associated with either clinical subphenotype) are found within the promoter GH11J027696 for *BDNF*. GeneHancer results for the variant rs10508649, associated with clinical subphenotypes, are described in section “Variants in *PIP4K2A.*” The GTEx database indicated that rs1053454 and rs2230469 (polymorphic in the cohort under study, but not associated with either clinical subphenotype) are expression quantitative trait loci (eQTLs) for *PIP4K2A* in various parts of the brain cortex and a number of subcortical nuclei. GTEx results for rs2248440 and rs6318 are described in section “**Variant in *HTR2C.***”

### Several Alleles Are Associated With Clinical Subphenotypes

Statistical tests indicated eight associations between alleles and clinical subphenotypes (Table 4). Two variants in *NRG1*, NC_000008.11:g.32614509_32614510del, and rs35641374, are associated with a number of subphenotypes related to depressive symptoms. Namely, the allele “del” of NC_000008.11:g.32614509_32614510del is associated with absence of response to antidepressant medication on the 28th day assessed by the HDRS-17 among patients with depression. Longer intervals between depressive episodes in the bipolar and cross-disorder groups are associated with the allele C of rs35641374. The same is for longer intervals between episodes of any type (i.e., depressive, manic or mixed) in the bipolar disorder group.

**TABLE 4:** Genetic variants, associated with clinical subphenotypes.

Chr	Pos (hg38)	Ref	Alt	Variant	Trait	Cohort	Model	Association	*p*-value	Gene	Strand	Variant biological function
8	32614509-32614510	TT	del	NC_000008.11: g.32614509_32614510del	Drug treatment response	Depression	Binomial logistic regression	Absence of response on the 28th day assessed by HDRS-17 is associated with allele “del”	3.22E-04	*NRG1*	F	Intronic, no obvious function
	32648114	G	C	rs35641374	Time to recurrence of a depressive episode	Cross disorder	Linear regression	Longer intervals between depressive episodes are associated with allele C	3.14E-06			Missense in five transcripts, Val > Leu, likely benign for protein function
					Time to recurrence of a depressive episode	Bipolar		Longer intervals between depressive episodes are associated with allele C	4.37E-07			
					Time to recurrence of an episode	Bipolar		Longer intervals between episodes of any type are associated with allele C	3.43E-04			
10	22541913	C	T	rs61731109	Drug treatment response	Depression	Binomial logistic regression	absence of response on the 28th day assessed by HDRS-17 is associated with allele T *(passed FDR, but not Bonferroni correction for multiple comparisons)*	1.11E-03	*PIP4K2A*	R	Synonymous in all transcripts; allele A (reverse complement) may affect splicing (^†^HumanSplicingFinder)
	22573353	T	C	rs10508649	Drug treatment response	Depression	Binomial logistic regression	Absence of response on the 28th day assessed by CGI-I scale is associated with allele C	9.43E-04			Synonymous in all transcripts; allele G (reverse compliment) may affect splicing (^†^HumanSplicingFinder); found within enhancer GH10J022572 that regulates expression of *PIP4K2A* (^†^GeneHancer)
					Time to recurrence of a manic or mixed episode	Bipolar	Linear regression	Longer intervals between manic or mixed episodes are associated with allele C	3.09E-04			
X	114727001	A*	G	rs2248440	Severity	Depression	Ordinal logistic regression	Higher severity of depression is associated with allele A	3.00E-03	*HTR2C*	F	Intronic; allele A decreases expression of *IL13RA2* in putamen (^†^GTEx)

Chr, chromosome; pos, position in the human genome; ref, reference allele; alt, alternate allele; ^†^used algorithms; * minor allele.

The allele A (in reverse complement) of rs61731109, found in *PIP4K2A*, is associated with absence of response to antidepressant medication on the 28th day assessed by HDRS-17 among patients with depression. This association passed FDR, but not Bonferroni correction for multiple comparisons. The allele G (in reverse complement) of rs10508649, located in the same gene, is associated with absence of response to antidepressant medication on the 28th day assessed by the CGI-I scale among patients with depression and with longer intervals between manic or mixed episodes among patients with bipolar disorder.

There was no association with response to antidepressant medication for different groups of antidepressants.

Finally, the allele A of rs2248440 (it is a reference allele that is in fact minor, ranging in frequency from 0 to 25% in all eight mentioned populations in dbSNP build 153), located in *HTR2C*, is associated with higher severity of depression among patients with depression.

### Further Analysis of Biological Function of Associated and Novel Variants

#### Functional Annotation of Associated Variants

##### Variants in NRG1

The variant NC_000008.11:g.32614509_32614510del, associated with response to antidepressant therapy assessed by the HDRS-17 is intronic, with no obvious biological function that could be predicted with bioinformatic tools used (Table 4). Although rs35641374, associated with time to recurrence of depression among bipolar and cross-disorder patients and with time to recurrence of episodes of any type among bipolar disorder patients, is a missense variant, it does not seem to affect protein function (both amino acids in the Val > Leu substitution have uncharged hydrophobic side chain).

##### Variants in PIP4K2A

The allele A (in reverse complement) of the synonymous variant rs61731109, associated with response to antidepressant therapy assessed by the HDRS-17, may affect splicing of introns flanking the constitutive exon 8 (ENST00000376573.9 and ENST00000545335.5) or 6 (ENST00000323883.11) of the *PIP4K2A*’s transcripts, by creating an exonic splicing silencer, as predicted by HumanSplicingFinder. The allele G (in reverse compliment) of another synonymous variant rs10508649, associated with response to antidepressant therapy assessed by the CGI-I scale, and with time to recurrence of a manic or mixed episode, may also affect splicing of introns flanking the constitutive exon 5 (ENST00000376573.9 and ENST00000545335.5) or 2 (ENST00000323883.11) of the *PIP4K2A*’s transcripts, by erasing an exonic splicing enhancer motif, as predicted by HumanSplicingFinder. Furthermore, this variant is found within the GeneHancer’s elite enhancer GH10J022572 that regulates the expression of *PIP4K2A*.

##### Variant in HTR2C

The allele A of the intronic variant rs2248440, associated with higher severity of depression, decreases the expression of the neighboring gene *IL13RA2* in the putamen, according to GTEx. The variant rs6318 also regulates the expression level of *IL13RA2* in the putamen; it is only polymorphic in the cohort under study but was previously associated with a number of phenotypes relevant to the pathogenesis of depression and to effects of psychotropic drugs (Drago and Serretti, 2009; Mickey et al., 2012; Brummett et al., 2014; Karanovic et al., 2015; Avery and Vrshek-Schallhorn, 2016; Chagraoui et al., 2016; Vyalova et al., 2017). The two variants are located 4325 bp apart and are in linkage disequilibrium (LD), according to LDlink^20^. *IL13RA2* is expressed in the brain, codes for the interleukin 13 receptor α2 subunit, and is found almost 94 kilobases (kb) downstream from *HTR2C*.

#### Functional Annotation of Novel Variants

The intronic variants NC_000003.12:g.119947358A > G, NC_000008.11:g.32614509_32614510del (associated with absence of response to antidepressant medication), NC_ 000008.11:g.32756363dup, NC_000008.11:g.32763214T > G, NC_000010.11:g.22539905_22539911del, and NC_000010.11:g. 22539924_22539937del, found in genes *GSK3B*, *NRG1*, and *PIP4K2A*, bear no obvious biological function as reported by the bioinformatic tools used.

The missense variant NC_000011.10:g.27658302T > C in *BDN*F creates the substitution Asp > Gly, i.e., a change from an acidic to uncharged hydrophobic amino acid. This change is deemed deleterious/damaging for the protein function by PROVEAN, PolyPhen-2, LRT, MutationTaster, and FATHMM-MKL algorithms run in ANNOVAR. The implicated allele G (in reverse compliment) may also affect splicing of the last intron of all *BDNF* transcripts, by erasing an exonic splicing enhancer motif, as predicted by HumanSplicingFinder. This variant is present in a heterozygous state in one patient with depressive episode of moderate severity and is not associated with clinical subphenotypes of mood disorders.

Finally, the 81 bp deletion in the last exon of all *HTR2C* transcripts, NC_000023.11:g.114906768_114906848del, creates an in-frame deletion of 27 amino acids (ENST00000276198.5 and ENST00000371951.5) or frameshift deletion of 27 amino acids, resulting in 1 aa inserted (ENST00000371950.3). The variant is deleterious for ENST00000276198.5 and ENST00000371951.5 according to PROVEAN (Choi and Chan, 2015). Moreover, it may affect splicing of the last intron of all *HTR2C* transcripts, by erasing multiple exonic splicing enhancer motifs, as deemed by HumanSplicingFinder. This deletion was present on the X chromosome in one male patient with recurrent depressive disorder of moderate severity. No association with clinical subphenotypes was established for that variant.

## Discussion

### Possible Genetic Biomarkers of Clinical Subphenotypes of Depression and Bipolar Disorder

#### Prognostic Biomarkers of Time to Recurrence of Mania and Depression in Bipolar Disorder

Time to recurrence of a depressive episode and time to recurrence of a manic or mixed episode among patients with bipolar disorder may be indicated by alleles of two different variants: rs35641374 in *NRG1* and rs10508649 in *PIP4K2A*, respectively (Table 4). The association between rs35641374 and time to recurrence of an episode of any type in the bipolar disorder group seems to be driven by the association between this genetic marker and time to recurrence of depression (the p-value increases from 4.37 × 10^–7^ for the latter association to 3.43 × 10^–4^). Likewise, the association signal at the same marker in the cross-disorder group may in fact be driven by the same association in the bipolar disorder group (the *p*-value increases from 4.37 × 10^–7^ for the latter association to 3.14 × 10^–6^). In other words, rs35641374 may be only associated with time to recurrence of a depressive episode among patients with bipolar disorder.

These results indicate that the allele C of rs35641374 located in *NRG1* and the allele G (reverse compliment) of rs10508649 located in *PIP4K2A* may be protective against recurrent depression and recurrent manic or mixed episodes, respectively. Although rs35641374 seems to be benign to the biological function of mRNA and protein, rs10508649 is found within the GeneHancer’s elite enhancer GH10J022572 that regulates the expression of *PIP4K2A*. Furthermore, the allele G of rs10508649 may affect mRNA splicing, which indicates that this variant may be functional for the *PIP4K2A* gene expression on two levels.

This conclusion suggests that *PIP4K2A* plays a role in the pathogenesis of manic or mixed symptoms. Previous data indicate that this gene is associated with bipolar disorder and schizophrenia (Stopkova et al., 2003; Schwab et al., 2006), both disorders sharing a number of clinical and molecular features (International Schizophrenia Consortium et al., 2009; Bipolar Disorder and Schizophrenia Working Group of the Psychiatric Genomics Consortium, 2018).

Implication of *NRG1* in the pathogenesis of depressive symptoms among patients with bipolar disorder is also supported by previous studies, linking this gene to bipolar disorder and depression (Mei and Nave, 2014; Dang et al., 2016; Wen et al., 2016; Chen et al., 2017).

#### Predictive Biomarkers of Response to Antidepressant Medication

Absence of response on the 28th day assessed by the HDRS-17 or CGI-I scale among patients with depression might be predicted by alleles of the variant NC_000008.11:g.32614509_32614510del in *NRG1* and of the two variants in *PIP4K2A*: rs61731109 and rs10508649. The indicated novel intronic variant should perhaps be listed under the dbSNP entry rs750640301 that differs from it only by the number of deleted nucleotides (one T instead of two) at the same genomic location. Although NC_000008.11:g.32614509_32614510del bears no obvious biological function, both rs61731109 and rs10508649 seem to affect splicing and, in case of rs10508649, transcriptional regulation of *PIP4K2A.*

#### Cross-Disorder Aspects of Mania and Antidepressant Therapeutic Response

It is worthy of note that the alleles of *NRG1* and *PIP4K2A* may be simultaneous predictors of time to recurrence of manic and depressive episodes among patients with bipolar disorder and of absence of drug treatment response among patients with depression (Table 4). In particular, the allele G (reverse compliment) of rs10508649 in *PIP4K2A* may increase resistance to antidepressant treatment and be at the same time protective against recurrent manic or mixed episodes. These data suggest interesting avenues for the study of the pathogenesis of mania and its possible connections with the pathogenesis of treatment-resistant depression (McIntyre et al., 2014). It is known that antidepressant drugs are not suitable in treatment of bipolar disorder, because they do not provide the desired response; moreover, evidence from clinical practice suggests that antidepressants may precipitate manic symptoms (Hirschfeld, 2014). It has even been suggested that switching to mania is a consequence of increased antidepressant efficacy (Lee H. J. et al., 2013), which would support the results implicating rs10508649. Both treatment-resistant depression and mania were also suggested to have one common underlying biological mechanism: circadian rhythms (Lee H. J. et al., 2013). Although *NRG1* and *PIP4K2A* are not regarded as biological clock genes, they both take part in signaling networks where *GSK3B* (Carter, 2007; Beaulieu, 2012), a gene implicated in circadian rhythms (Harada et al., 2005), plays a prominent role. An additional clue in this puzzle is that lithium, which acts upon this pathway and inhibits GSK3B (Freland and Beaulieu, 2012), modulates circadian rhythms in patients with bipolar disorder (Lee H. J. et al., 2013). Further research is necessary to continue uncovering the complex interplay between molecular networks involving GSK3B, circadian rhythms, manic symptoms, and antidepressant resistance.

#### Prognostic Biomarker of Depression Severity

Depression severity in patients with a diagnosis of depression may be determined by alleles of the variant rs2248440 found in the intron following the first coding exon of *HTR2C*. This variant is in LD with the missense *HTR2C* variant rs6318, previously associated with mood disorders and response to antidepressant medication (Chagraoui et al., 2016; Vyalova et al., 2017). An interesting finding is that both variants are eQTLs in the putamen for the gene of a subunit of the interleukin 13 (IL-13) receptor (*IL13RA2*). This might indicate either that the serotonin 2C receptor is not a genetic factor influencing on severity of depressive symptoms or that both receptors act in concert in influencing on this clinical subphenotype. Knowing that the involvement of the serotonin 2C receptor in the pathogenesis of depression is supported by a considerable amount of scientific data (Chagraoui et al., 2016; Palacios et al., 2017) and that the pathogenesis of depression is associated with the immune system abnormalities (Felger and Lotrich, 2013), the latter scenario seems to be more plausible. Although *IL13RA2* is known to be implicated mainly in cancer (Sengupta et al., 2014), its ligand IL-13 regulates inflammation (Mao et al., 2019), so this gene could also play a part in increased inflammation seen in patients with depression. More research is needed to clarify these data.

#### Estimation of Sample Size

Estimation of sample size is possible, but not trivial, because it necessitates prior knowledge of relative risks and frequencies of genotypes associated with the disease. These parameters are unavailable in the case of genetic variants that were not studied previously. It is also worthy of note that application of algorithms that can calculate sample size for genetic association studies is limited in the case of complex study designs. For example, Genetic Power Calculator^21^ can be used only for discrete traits. Application of this calculator, assuming that disease prevalence = 0.05, disease allele frequency = 0.05, and genotype relative risk = 2, indicates a sample size of 676 needed to achieve power of 80% for an allelic test at α = 0.05. However, our study design also includes quantitative traits. The instrument Power for Genetic Association Analyses^22^ (Menashe et al., 2008) does not include allelic tests. Nevertheless, assuming the dominant mode of inheritance, this statistical instrument indicates approximately the same sample size under the same parameters. General sample size calculators (such as^23^) indicate a sample size of 385 at α = 0.05, assuming that the actual population is very large (more than 1 million).

Power refers to the number of patients required to avoid a type II error (false-negative results) in a comparative study. Sample size estimation indicates that power of the present study is low. Nevertheless, the study showed a number of statistically significant results, probably due to detailed phenotyping that enabled using a smaller subset of patients (Yehia and Eng, 2019).

### Protein Networks

#### Interactions Suggested by This Study

NRG1 and PIP4K2A could act in concert via their connections with GSK3B (Figure 1; Carter, 2007; Beaulieu, 2012; Mei and Nave, 2014; Jiang et al., 2015; Greene and Hoshi, 2017). ERBB4 and its ligand NRG1 play an important role in neurodevelopment, neurotransmission, and synaptic plasticity, and this receptor is present on GABAergic, glutamatergic and dopaminergic neurons (Mei and Nave, 2014). One of its functions is to promote the inhibitory GABA release. Thus, together with KCNQ/M-channels activated by PIP2 (Greene and Hoshi, 2017), ERBB4 takes part in regulation of neuronal excitability, an aspect that could be important in the pathogenesis of bipolar disorder (Berridge, 2014).

Serotonin acts upon the brain and peripheral immune system components, while both types of these components regulate the serotonin neurotransmission (Robson et al., 2017; Wu et al., 2019). This connection could be the key to the observed immune system abnormalities strongly associated with depression (Felger and Lotrich, 2013) and suggests a possibility of an interaction between HTR2C and IL13RA2. The immune system could therefore be a biological factor that modulates the severity of depression (Felger and Lotrich, 2013).

On the other hand, knowing that serotonin leads to inhibition of KCNQ/M-channels (Colino and Halliwell, 1987; Stephens et al., 2018) and inflammation leads to production of reactive oxygen species resulting in increased neuronal excitability (Berridge, 2014), NRG1, PIP4K2A, HTR2C, and IL13RA2 may have further interconnected roles in the pathophysiology of depressive and manic episodes and the response to antidepressant medication.

#### Interactions Predicted by the String Database Relevant to This Study

Generalized protein networks may be visualized with String V.11 (Szklarczyk et al., 2019). In fact, the protein products of the genes described in this study – *GSK3B*, *BDNF*, *NRG1*, *NGF*, *HTR2C*, *PIP4K2A, IL13RA2*, *IL13*, *ERBB4*, *PIK3CA*, *PIK3CB*, *PDPK1*, *AKT1*, the three M-channel subunits *KCNQ2*, *KCNQ3*, and *KCNQ5*, and neurotrophic receptors *NTRK1* and *NTRK2* – are deemed to be functionally connected (Supplementary Figure S1A). Adding top 20 predicted functional partners to this network within the first shell of interactions (a direct connection with the input proteins) reveals a larger network (Supplementary Figure S1B).

It is important to note that predicted interactions should be interpreted with caution: bioinformatic instruments that predict interaction networks (such as String) cannot extract *all* connections relevant in psychiatric disorders, not they can draw protein networks relevant *only* in psychiatric disorders. For example, despite evidence in literature indicating regulation of KCNQ2 by GSK3B (Carter, 2007; Wildburger and Laezza, 2012; Jiang et al., 2015; Figure 1), no such interaction was predicted by String (Supplementary Figures S1A,B).

Despite this, predicted interactions may be used to pinpoint interesting functional candidates that may become subjects of future studies. For instance, String indicates a functional connection between HTR2C and IL13RA2 through the interleukin 4 receptor subunit α (IL-4R). A heterodimer formed by this subunit and the interleukin 13 receptor subunit α1 binds both IL-13 and IL-4 (Karo-Atar et al., 2018). IL-4 is known to be implicated in psychiatric disorders, in particular, in the pathogenesis of depression (Wachholz et al., 2017). One of IL-4’s roles is regulation of the serotonin transporter (Mossner et al., 2001). *PPP2CA* codes for the protein phosphatase 2 catalytic subunit α of protein phosphatase 2A (PP2A), an important component of AKT/GSK3 signaling implicated in a number of psychiatric disorders, including depression and bipolar disorder (Beaulieu, 2012), as well as response to psychotropic drugs, including antidepressants and lithium (Beaulieu et al., 2009). Protein products of *MTOR* and *TSC2* are implicated via their connections with AKT1 in regulation of synaptic plasticity and memory (Emamian, 2012); these are impaired in depression, possibly as a result of a reduction of hippocampal volumes (MacQueen and Frodl, 2011). As mentioned earlier, *PIK3R1* and *PIK3R2* code for regulatory subunits p85α and p85β, while *PIK3CA* codes for the catalytic subunit p110α of PI3K; expression levels of these subunits were significantly altered in suicide completers (Dwivedi et al., 2008). Suicide is a psychiatric phenotype associated with depression (Mullins et al., 2019), so pathogenic mechanisms may be shared between the two phenotypes. Finally, β-catenin encoded by *CTNNB1* is implicated in a number of molecular pathways relevant in psychiatric disorders (Freyberg et al., 2010; Wisniewska, 2013). *CTNNB1* also contains damaging genetic variants in several neurodevelopmental disorders, including schizophrenia (Levchenko et al., 2015), while some antipsychotic drugs result in increased levels of β-catenin in the brain (Freyberg et al., 2010). Bipolar disorder and schizophrenia share pathogenic mechanisms (Gordovez and McMahon, 2020), so β-catenin might be relevant in the pathogenesis of bipolar disorder as well (Guo et al., 2019).

### Study Limitations

The main limitation of this genetic study is a modest number of patients, which is reflected in reduced statistical power. This might have been the reason several interesting (from the point of view of predicted biological function) genetic candidates failed to show an association with clinical subphenotypes. Among these variants are rs66866077 and rs79679324 (that may modify the *BDNF* promoter activity), as well as rs1053454 and rs2230469 (that determine the *PIP4K2A* level of expression in various parts of the brain). The same may be noted about two novel, apparently deleterious variants NC_000011.10:g.27658302T > C (*BDNF*) and NC_000023.11:g.114906768_114906848del (*HTR2C*). Larger cohorts are necessary in order to investigate significance of these putative functional variants.

Another limitation is that any statistics-based study only points out avenues for further research, so in order to prove biological relevance of candidate genetic biomarkers, described in this paper, functional studies deploying cellular or animal models are warranted.

## Data Availability Statement

The datasets presented in this article are available at dbSNP: https://www.ncbi.nlm.nih.gov/projects/SNP/snp_ss.cgi?subsnp_ id=ss2137544101, https://www.ncbi.nlm.nih.gov/projects/SNP/snp_ss.cgi?subsnp_id=ss3986007706, https://www.ncbi.nlm.nih.gov/projects/SNP/snp_ss.cgi?subsnp_id=ss3986007707, https://www.ncbi.nlm.nih.gov/projects/SNP/snp_ss.cgi?subsnp_id=ss3986007708, https://www.ncbi.nlm.nih.gov/projects/SNP/snp_ss. cgi?subsnp_id=ss3986007709, https://www.ncbi.nlm.nih.gov/projects/SNP/snp_ss.cgi?subsnp_id=ss3986007710, https://www. ncbi.nlm.nih.gov/projects/SNP/snp_ss.cgi?subsnp_id=ss398600 7711, and https://www.ncbi.nlm.nih.gov/dbvar/studies/nstd180.

## Ethics Statement

The studies involving human participants were reviewed and approved by the Local Bioethics Committee of the Mental Health Research Institute in Tomsk, Russia. The patients/participants provided their written informed consent to participate in this study.

## Author Contributions

NV, NB, and SI contributed to the conception and design of the study. GS was the psychiatrist who recruited and clinically assessed the patients. SI recruited the controls. GS and NV built the clinical database. NV and IP performed the sequencing. TN performed the sequencing analysis, association study, and a part of functional annotation. AL supervised the work of TN, performed the remaining part of functional annotation, integrated the study results, and wrote the manuscript. All authors contributed to the manuscript revision and read and approved the submitted version.

## Conflict of Interest

The authors declare that the research was conducted in the absence of any commercial or financial relationships that could be construed as a potential conflict of interest. The reviewer, AK, declared a shared affiliation, with no collaboration, with one of the authors, AL, to the handling editor at the time of review.

## Footnotes

1 https://www.ampliseq.com

2 https://broadinstitute.github.io/picard/

3 http://www.bioinformatics.babraham.ac.uk/projects/fastqc/

4 http://bio-bwa.sourceforge.net/

5 https://software.broadinstitute.org/gatk/documentation/tooldocs/4.1.2.0/

6 http://genome.ucsc.edu/cgi-bin/hgLiftOver

7 https://www.ncbi.nlm.nih.gov/snp/

8 http://exac.broadinstitute.org/

9 https://gnomad.broadinstitute.org/

10 https://software.broadinstitute.org/gatk/

11 https://www.cog-genomics.org/plink/2.0/

12 https://gtexportal.org

13 https://www.rdocumentation.org/packages/SNPRelate

14 https://www.rdocumentation.org/packages/dplyr

15 https://www.rdocumentation.org/packages/GenABEL

16 https://www.rdocumentation.org/packages/ggplot2

17 https://www.rdocumentation.org/packages/manhattanly

18 https://www.rdocumentation.org/packages/GWASTools

19 https://www.rdocumentation.org/packages/GENESIS

20 https://ldlink.nci.nih.gov/

21 http://zzz.bwh.harvard.edu/gpc/

22 https://dceg.cancer.gov/tools/design/pga

23 https://www.surveymonkey.com/mp/sample-size-calculator/

## Supplementary Material

FIGURE S1 Shown are molecular interactions as predicted by String. **(A)** A network limited only to input proteins. In evidence mode in String, an edge is drawn with differently colored lines that represent the existence of various types of evidence used in predicting the associations. Blue line – cooccurrence evidence, purple line – experimental evidence, yellow line – textmining evidence (i.e., the two proteins are co-mentioned in PubMed abstracts), light blue line – database evidence, and black line – coexpression evidence. **(B)** The same network expanded with additional top 20 predicted functional partners within the first shell of interactions (a direct connection with the input proteins). In confidence mode in String the thickness of the line indicate the degree of confidence prediction of the interaction.
