# Supplementary figures and images for: NRG1, PIP4K2A, and HTR2C as Potential Candidate Biomarker Genes for Several Clinical Subphenotypes of Depression and Bipolar Disorder
Source: Front Genet. 2020 Aug 25;11:936. doi: 10.3389/fgene.2020.00936 (PMC7478333; doi:10.3389/fgene.2020.00936)

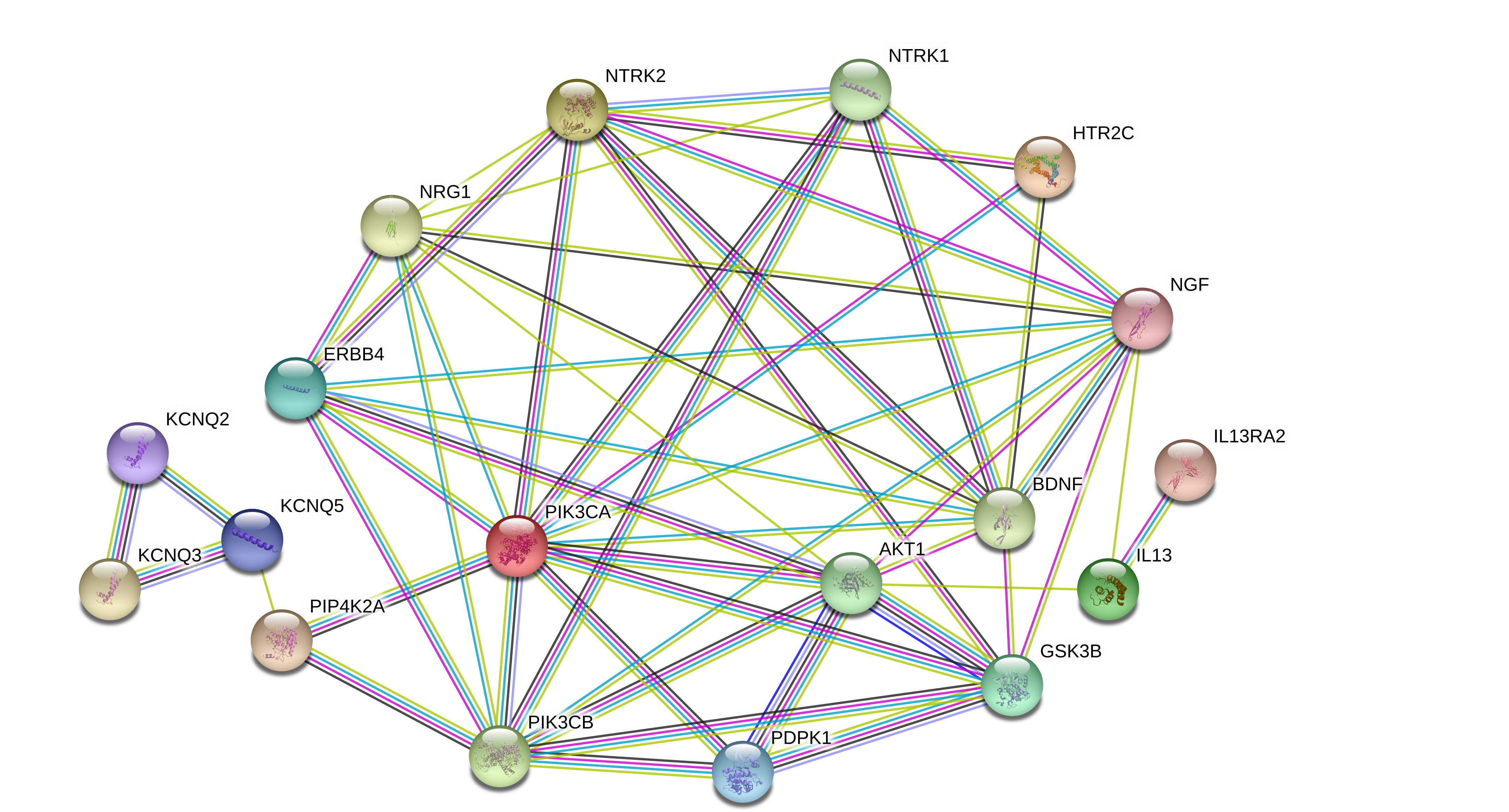

Supplement: FIGURE S1 — Shown are molecular interactions as predicted by String. (A) A network limited only to input proteins. In evidence mode in String, an edge is drawn with differently colored lines that represent the existence of various types of evidence used in predicting the associations. Blue line – cooccurrence evidence, purple line – experimental evidence, yellow line – textmining evidence (i.e., the two proteins are co-mentioned in PubMed abstracts), light blue line – database evidence, and black line – coexpression evidence. (B) The same network expanded with additional top 20 predicted functional partners within the first shell of interactions (a direct connection with the input proteins). In confidence mode in String the thickness of the line indicate the degree of confidence prediction of the interaction. [file Presentation_1.zip › Image 1A.TIF]

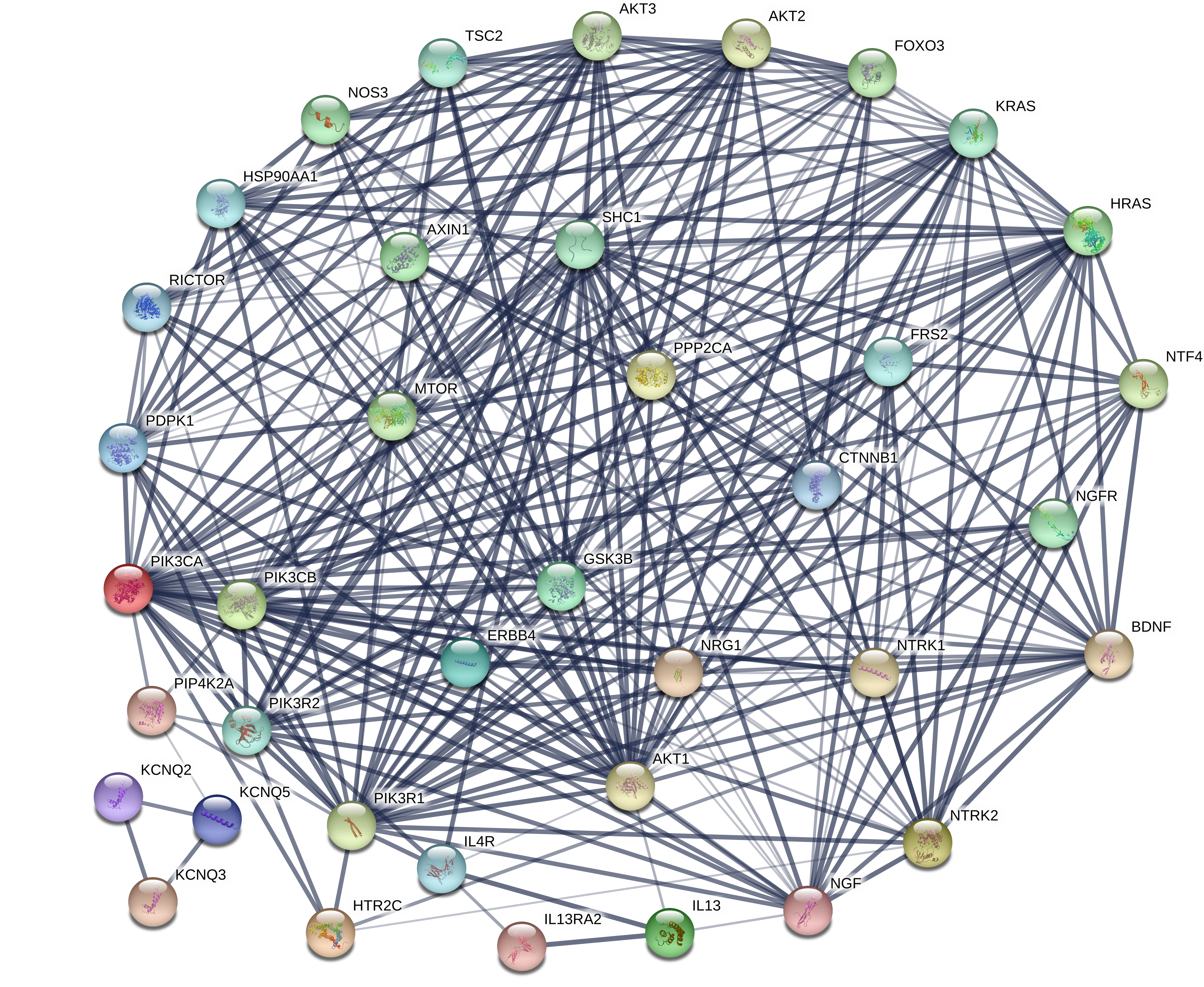

Supplement: FIGURE S1 — Shown are molecular interactions as predicted by String. (A) A network limited only to input proteins. In evidence mode in String, an edge is drawn with differently colored lines that represent the existence of various types of evidence used in predicting the associations. Blue line – cooccurrence evidence, purple line – experimental evidence, yellow line – textmining evidence (i.e., the two proteins are co-mentioned in PubMed abstracts), light blue line – database evidence, and black line – coexpression evidence. (B) The same network expanded with additional top 20 predicted functional partners within the first shell of interactions (a direct connection with the input proteins). In confidence mode in String the thickness of the line indicate the degree of confidence prediction of the interaction. [file Presentation_1.zip › Image 1B.TIF]
